# Synthesis, Spectroscopic, and Antimicrobial Studies on Bivalent Nickel and Copper Complexes of Bis(thiosemicrbazone)

**DOI:** 10.1155/2007/51483

**Published:** 2007-09-27

**Authors:** Sulekh Chandra, Smriti Raizada, Monika Tyagi, Archana Gautam

**Affiliations:** ^1^Department of Chemistry, Zakir Husain College, University of Delhi, Jawaharlal Nehru Marg, New Delhi 110002, India; ^2^Department of Chemistry, MMH College Ghaziabad, 201001, India

## Abstract

A series of metal complexes of Cu(II) and Ni(II) having the general composition $\left[\text{M}\left(\text{L}\right){\text{X}}_{2}\right]$ with benzil bis(thiosemicarbazone) has been prepared and characterized by element chemical analysis, molar conductance, magnetic susceptibility measurements, and spectral (electronic, IR, EPR, mass) studies. The IR spectral data suggest the involvement of sulphur and azomethane nitrogen in coordination to the central metal ion. On the basis of spectral studies, an octahedral geometry has been assigned for Ni(II) complexes but a tetragonal geometry for Cu(II) complexes. The free ligand and its metal complexes have been tested in vitro against a number of microorganisms in order to assess their antimicrobial properties.

## 1. INTRODUCTION

The chemistry of thiosemicarbazones has received considerable attention in view of their variable bonding modes, promising biological implications, structural diversity, and ion-sensing ability [1–3]. They have been used as drugs and are reported to possess a wide variety of biological activities against bacteria, fungi, and certain type of tumors and they are also a useful model for bioinorganic processes [4, 5]. As regards biological implications, thiosemicarbazone complexes have been intensively investigated for antiviral, anticancer, antitumoral, antimicrobial, antiamoebic, and anti-inflammatory activities. The inhibitory action is attributed due to their chelating properties [6–16]. The activity of these compounds is strongly dependent upon the nature of the heteroatomic ring and the position of attachment to the ring as well as the form of thiosemicarbazone moiety [17]. These are studied extensively due to their flexibility, their selectivity and sensitivity towards the central metal atom, structural and similarities with natural biological substances, due to the presence of imine group (−N=CH−) which imparts the biological activity [18].

**Figure 1:**
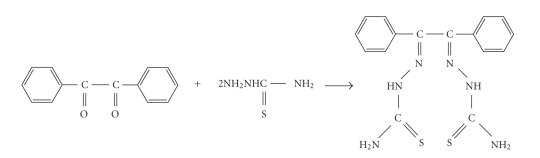
Synthesis and structure of ligand.

In view of the above applications, the present work relates to the synthesis, spectroscopic, and antimicrobial studies of Cu(II) and Ni(II) complexes with benzil bis(thiosemicarbazone). The ligand used in the study is depicted in Figure 1.

## 2. EXPERIMENTAL

### 2.1. Materials

All the chemicals used were of Anala R grade and procured from Sigma-Aldrich and Fluka. Metal salts were purchased from E. Merck and used as received.

### 2.2. Synthesis of ligand(L)

Hot ethanolic solution of thiosemicarbazide (1.82 g, 0.02 mol) and ethanolic solution of benzil (2.1 g, 0.01 mol) were mixed in the presence of few drops of conc.HCl with constant stirring. This mixture was refluxed at 60–70°C for 3 hours. The completion of the reaction was confirmed by the TLC. The reaction mass was degassed on a rotatory evaporator, over a water bath. The degassed reaction mass on cooling gives cream-colored crystals. It was filtered, washed with cold EtOH, and dried under vacuum over P_4_O_10_, (yield (65%), mp 164°C). Element chemical analysis data are shown in Table 1.

### 2.3. Synthesis of complexes

Hot ethanolic solution (20 mL) of corresponding metal salts (0.01 mol) was mixed with hot ethanolic solution of the respective ligand (0.01 mol). The mixture was refluxed for 3-4 hours at 50–60°C. On cooling the contents, the colored complex separated out in each case. It was filtered and washed with 50% ethanol and dried under vacuum over P_4_O_10_. Purity of the complexes was checked by TLC.

### 2.4. Analysis

The C, H, and N were analyzed on Carlo-Erba 1106 elemental analyzer. The Nitrogen content of the complexes was determined using Kjeldahl's method. Molar conductance was measured on the ELICO (CM82T) conductivity bridge. Magnetic susceptibilities were measured at room temperature on a Gouy balance using CuSO_4_·5H_2_O as callibrant. Diamagnetic corrections were made by using Pascal's constants. Electronic impact mass spectrum was recorded on Jeol, JMS - DX-303 mass spectrometer. IR spectra (KBr) were recorded on FTIR spectrum BX-II spectrophotometer. The electronic spectra were recorded in DMSO on Shimadzu UV mini-1240 spectrophotometer. EPR spectra of the Cu(II) complexes were recorded as polycrystalline sample at room temperature E_4_-EPR spectrometer using the DPPH as the g-marker. The molecular weights of complexes were determined cryoscopically in benzene.

### 2.5. Antibacterial screening

The antibacterial activity of the ligand and its metal complexes were tested by using paper disc diffusion method [19–21] against *Bacillus macerans* (gram-positive) and *Pseudomonas striata* (gram-negative). Nutrient agar medium was prepared by using peptone, beef extract, NaCl, agar-agar, and distilled water. The test compounds in measured quantities were dissolved in DMF to get concentrations of 250, 125, and 63.5 ppm of compounds. Twenty five millileter nutrient agar media (NA) was poured in each Petri plates. After solidification, 0.1 mL of test bacteria spread over the medium using a spreader. The discs of Whatmann no. 1 filter paper having the diameter 5.00 mm, each containing 1.5 mg cm^−1^ of compounds, were placed at four equidistant places at a distance of 2 cm from the center in the inoculated Petri plates. Filter paper disc treated with DMF served as control and Streptomycin used as a standard drug. All determination was made in duplicate for each of the compounds. An average of two independent readings for each compound was recorded. These Petriplates were kept in refrigerator for 24 hours for prediffusion. Finally, Petri plates were incubated for 26–30 hours 28±2°C. The zone of inhibition was calculated in millimeters carefully.

### 2.6. Antifungal screening

The preliminary fungitoxicity screening of the compounds at different concentrations was performed in vitro against the test fungi, *R.bataticola*, *A.alternata* and *F.Odum* by the food poision technique [22, 23]. Stock solutions of compounds were prepared by dissolving the compounds in DMF. Chlorothalonil was used as a commercial fungicide and DMF served as a means of control. Potato dextrose agar medium was prepared by using potato, dextrose, agar-agar, and distilled water. Appropriate quantities of the compounds in DMF were added to potato dextrose agar medium in order to get concentrations of 250, 125, 62.5 ppm of compound in the medium. The medium was poured into a set of two Petri plates under aseptic conditions in a laminar flow hood. When the medium in the plates was solidified, mycelial discs of 0.5 cm in diameter-cut from the periphery of the 7-day old culture and were aseptically inoculated upside down in the centre of the Petri plates. These treated Petri plates were incubated at 26±1°C until fungal growth in the control Petriplates was almost complete.

The mycelial growth of fungi (mm) in each petriplate was measured diametrically and growth inhibition (I) was calculated using the formula

(1) $$\text{I}\left(\text{\%}\right)=\frac{\left(\text{C-T}\right)}{\text{C}}\times \text{100,}\text{​}\;\;\;\;\;\;\text{IC}=\left[\frac{\left(\text{I-CF}\right)}{\text{100CF}}\right]\times 100,$$

where CF = (90-Co)/x 100, 90 is the diameter (mm) of the petri plates, and Co is the growth of the fungus (mm) in control.

## 3. RESULTS AND DISCUSSION

**Figure 2:**
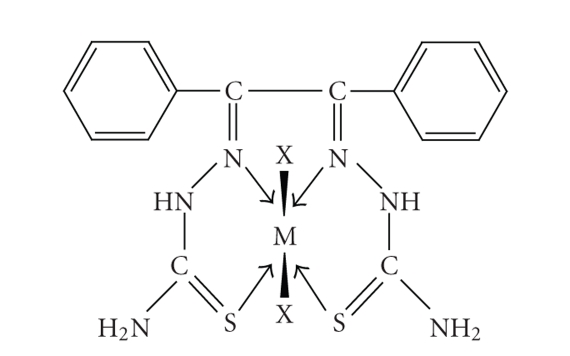
Suggested structure of the complexes.

The complexes were synthesized by reacting ligand with the metal ions in 1 : 1 molar ratio in ethanolic medium. The ligand behaves as tetradentate coordinate through sulphur and nitrogen donor atoms (Figure 2). All the nickel(II) and copper(II) complexes are paramagnetic in nature. The analytical data, magnetic susceptibility, and spectral analysis agree well with the proposed composition of formed complexes. All the complexes have shown good solubility in all the common organic solvents, but they were found insoluble in ether, water, acetone, and benzene. The molar conductance of the complexes in DMF lies in the range of 10–20 Ω^−1^cm^2^mol^−1^ indicating their nonelectrolytic behavior. Thus, the complexes may be formulated as [M(L)X_2_] (where M = Ni(II), Cu(II); L = benzil bis(thiosemicarbazone); X = Cl^−^, NO_3_^−^, and CH_3_COO^−^).

## 4. MASS SPECTRUM

**Figure 3:**
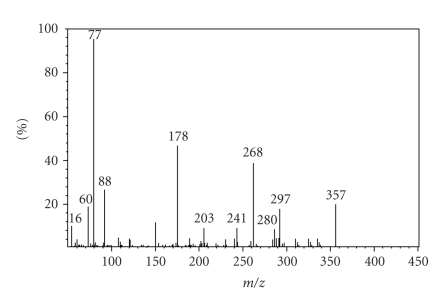
Electronic impact mass spectrum of ligand (L).

The electronic impact mass spectrum of the ligand (Figure 3) shows a molecular ion (M^+^) peak at m/z = 357 amu corresponding to species [C_16_H_16_N_4_S_2_]^+^, which confirms the proposed formula. It also shows series of peaks at 16, 60, 77, 88, 178, 203, 241, 268, 280, and 297 amu, corresponding to various fragements. The intensities of these peaks give the idea of the stabilities of the fragements.

## 5. MAGNETIC SUSCEPTIBILITY

The observed magnetic moments of Ni(II) and Cu(II) complexes are given in Table 1. The best summary of the results on the magnetic behavior of nickel and copper compounds was given by Figgis and Nyholm [24]. The observed values of magnetic moment for complexes are generally diagnostic of the coordination geometry about the metal ion. Ni(II) has the electronic configuration 3d^8^ and should exhibit a magnetic moment higher than that expected for two unpaired electrons in octahedral (2.8–3.2 BM) and tetrahedral (3.4–4.2 BM) complexes, whereas its square planar complexes would be diamagnetic. The magnetic moment observed for the Ni(II) complexes lies in the range of 2.89–2.95 BM which is consistent with the octahedral stereochemistry of the complexes. Room-temperatue magnetic moment of the Cu(II) complexes lies in the range of 1.92–1.98 BM, corresponding to one unpaired electron. Whatsoever the geometry of Cu(II) is, its complexes always show magnetic moment corresponding to one unpaired electron.

## 6. INFRARED SPECTRA

The assignments of the significant IR spectral bands of ligand and its metal complexes are presented in Table 2. In principle, the ligand can exhibit thione-thiol tautomerism since it contains a thioamide−NH−C = S functional group. The *ν*(S−H) band at 2565 cm^−1^ is absent in the IR spectrum of ligand but *ν*(N−H) band at ca.3237 cm^−1^ is present, indicating that in the solid state, the ligand remains as the thione tautomer. The position of *ν*(C = N) band of the thiosemicarbazone appeared at 1608 cm^−1^ is shifted towards lower wave number in the complexes indicating coordination via the azomethane nitrogen [25, 26]. This is also confirmed by the appearance of bands in the range of 459–485 cm^−1^, this has been assigned to the *ν*(M−N) [27]. A strong band found at 1106 cm^−1^ is due to the *ν*(N−N) group of the thiosemicarbazone. The position of this band is shifted towards higher wave number in the spectra of complexes. It is due to the increase in the bond strength, which again confirms the coordination via the azomethane nitrogen. The band appearing at ca. 837 cm^−1^ *ν*(C = S) in the IR spectrum of ligand is shifted towards lower wave number. It indicates that thione sulphur coordinates to the metal ion [28]. Thus, it may be concluded that the ligand behaves as tetradentate chelating agent coordinating through azomethane nitrogen and thiolate sulphur [29].

## 7. ANIONS

The presence of bands at 1457-1412, 1320-1299, and 1078-1012 cm^−1^, in the IR spectra of the metal complexes of Ni(II) and Cu(II), suggests that both nitrate groups are coordinated to the central metal ion in a unidentate fashion. In the IR spectra of chloro complexes, bands corresponding to *ν*(M−Cl) are observed at 345-320 cm^−1^indicating the presence of M−Cl bond. The IR spectra of Ni(II) and Cu(II) of acetato complexes show the medium intensity bands at 1620-1619 and 1332-1321 cm^−1^, assigned to *ν*_a_(C−O) and *ν*_s_(C−O), respectively. The difference between these two frequencies is ∼287 cm^−1^, which is greater than that for uncoordinated acetate ion by ∼143 cm^−1^ and that for bidentate acetate ion by ∼217 cm^−1^. It is strongly supported that both acetate ions are coordinated to the metal ion in a unidentate fashion [30–32].

## 8. ELECTRONIC SPECTRA

**Nickel(II) complexes**

The electronic spectra of Ni(II) complexes display three absorption bands (Table 3) in the ranges of 9870-9337 cm^−1^, 14577-14124 cm^−1^, and 25700-24100 cm^−1^. The ground state nickel(II) in an octahedral coordination is ^3^A_2g_. Thus, these bands may be assigned to three spin-allowed transitions: ^3^A_2g_(F) → ^3^T_2g_(F)(*ν*_1_), ^3^A_2g_(F) → 3T_1g_(F)(*ν*_2_), and ^3^A_2g_ (F) → ^3^T_1g_(P) (*ν*_3_), respectively. The position of bands indicates that the complexes have six coordinated octahedral geometries [33]. Various ligand field parameters were calculated for the Ni(II) complexes and listed in Table 3. The values of Dq and B were calculated by using Orgel diagram. The ratio *ν*_1_/*ν*_2_ was considered for the calculation of B. The Nephelauxetic parameter *β* was readily obtained by using the relation: *β* = B(complex)/B(free ion), where B(free ion ) for Ni(II) is 1041 cm^−1^. The *β* values lying in the range of 0.58–0.61 indicate the appreciable covalent character of metal ligand “*σ*” bond [34].

**Copper(II) complexes**

The electronic spectra of Cu(II) complexes display bands in the ranges of 15432-14727 cm^−1^ and 25575-25380 cm^−1^ (Table 3). These bands correspond to the transitions ^2^B_1g_ → ^2^A_1g_(d_x2−y2_ → d_z2_)*ν*_1_ and ^2^B_1g_ → ^2^B_2g_(d_x2-y2_ → d_zy_)*ν*_2_, respectively. The third band in the range of 33670-32570 cm^−1^ may be due to charge transfer. Therefore, the complexes may be considered to possess a tetragonal geometry [35, 36].

## 9. ELECTRONIC PARAMAGNETIC SPECTRA

Room-temperature EPR spectra of Cu(II) complexes were recorded as polycrystalline sample, on X band at frequency of 9.1 GHz under the magnetic-field strength of 3000G. The analysis of spectra gives ${\text{g}}_{∥}=-2.25-2.10,\;\;{\text{g}}_{⊥}=2.14-2.03$ (Table 4). The observed ${\text{g}}_{∥}$ values for the complexes are less than 2.3 in agreement with the covalent character of the metal ligand bond. The trend ${\text{g}}_{∥}>{\text{g}}_{|}>2.0023$ observed for the complexes indicates that unpaired electron is localized in d_x2-y2_ orbital of the Cu(II) ion and the spectral features are a characteristic of axial symmetry. Thus, a tetragonal geometry is confirmed for the aforesaid complexes [37].

$\text{G}=\left({\text{g}}_{∥}-2\right)/\left({\text{g}}_{⊥}-2\right)$, which measures the exchange interaction between the metal centres in a polycrystalline solid, has been calculated. According to Hathaway [38] if G > 4, the exchange interaction is negligible, but G < 4 indicates considerable exchange interaction in the solid complexes. The complexes reported in this paper, given the “G” value, are < 4 indicating the exchange interaction in solid complexes.

## 10. ANTIMICROBIAL STUDIES

The antimicrobial screening data show that the compounds exhibit antimicrobial properties, and it is important to note that the metal chelates exhibit more inhibitory effects than the parent ligands. From Table 5 it is clear that the zone of inhibition is much larger for metal complexes against the gram-positive bacteria (*Bacillus macerans*) and gram-negative bacteria (*Pseudomonas striata*). The increased activity of the metal chelates can be explained on the basis of chelation theory [39]. It is known that chelation tends to make the ligand act as more powerful and potent bactericidal agents, thus killing more of the bacteria than the ligand. It is observed that, in a complex, the positive charge of the metal is partially shared with the donor atoms present in the ligands, and there may be *π*-electron delocalization over the whole chelating [39]. This increases the lipophilic character of the metal chelate and favours its permeation through the lipoid layer of the bacterial memberanes.There are other factors which also increase the activity, which are solubility, conductivity, and bond length between the metal and the ligand.

The results of fungicidal screening (Table 6) show that Cu(II) and Ni(II) complexes were highly active than the free ligand against phytopathogenic fungi, *Rhizoctonia bataticola*, *Alternaria alternata*, and *Fusarium odum*. The mode of action may involve the formation of a hydrogen bond through the azomethane nitrogen atom with the active centers of the cell constituents, resulting in interference with the normal cell process.The variation in the effectiveness of different compounds against different organisms depends either on the impermeability of the cells of the microbes or the difference in ribosomes of microbial cells [40]. It has also been proposed that concentration plays a vital role in increasing the degree of inhibition; as the concentration increases, the activity increases.

## Figures and Tables

**Table 1:** Analytical data for the ligand and its Ni(II) and Cu(II) complexes.

Compounds	Atomic mass found (calcd.)	Yield (%)	Color	Mp (°C)	Analysis found (calcd.)				$\mu_{\text{eff}}\;\text{BM}$
					C	H	N	M	
C_16_H_16_N_6_S_2_ ligand (L)	357 (356)	65	Cream	164	53.91 (53.93)	4.45 (4.49)	23.56 (23.60)	—	—
[Ni(L)Cl_2_]	487 (486)	66	Brown	282	39.48 (39.51)	3.26 (3.29)	17.29 (17.28)	12.10 (12.08)	2.89
[Ni(L)(NO_3_)_2_]	538 (539)	70	Dark brown	290	35.64 (35.62)	2.98 (2.97)	20.75 (20.78)	10.85 (10.89)	2.95
[Ni(L)(CH_3_COO)_2_]	535 (533)	68	Brown	285	45.04 (45.03)	4.12 (4.13)	15.74 (15.76)	11.03 (11.01)	2.92
[Cu(L)Cl_2_]	492 (491)	72	Green	176	39.08 (39.10)	3.24 (3.26)	17.14 (17.11)	12.95 (12.93)	1.95
[Cu(L)(NO_3_)_2_]	546 (544)	66	Green	180	35.26 (35.29)	2.92 (2.94)	20.57 (20.59)	11.62 (11.67)	1.92
[Cu(L)(CH_3_COO)_2_]	539 (538)	64	Light green	185	44.58 (44.60)	4.06 (4.09)	15.63 (15.61)	11.87 (11.80)	1.98

**Table 2:** Important infrared spectral bands (cm^−1^) and their assignments.

Compounds	Assignements				
	*ν*(N−H)	*ν*(N−N)	*N*(C*=*N)	*ν*(C*=*S)	*ν*(M−N)
Ligand (L)	3237	1106	1608	837	—
[Ni(L)Cl_2_]	3260	1125	1570	816	479
[Ni(L)(NO_3_)_2_]	3272	1128	1595	825	465
[Ni(L)(CH_3_COO)_2_]	3255	1123	1585	815	459
[Cu(L)_2_Cl_2_]	3250	1124	1560	810	485
[Cu(L)(NO_3_)_2_]	3261	1123	1596	818	460
[Cu(L)_2_(CH_3_COO)_2_]	3264	1125	1590	820	475

**Table 3:** Electronic spectral bands (cm^−1^) and ligand field parameters of the complexes.

Complex	$\gamma_{\max}\left({\text{cm}}^{-1}\right)$	*ε*(Lmol^−1^cm^−1^)	*ν*_2_/*ν*_1_	Dq (cm^−1^)	*B*(cm^−1^)	*β*
[Ni(L)Cl_2_]	9337, 14124, 24100	30, 48, 60	1.5	1018	599	0.58
[Ni(L)(NO_3_)_2_]	9670, 14388, 24570	32, 50, 61	1.5	1054	620	0.60
[Ni(L)(CH_3_COO)_2_]	9870, 14577, 25700	32, 52, 63	1.5	1076	632	0.61
[Cu(L)Cl_2_]	14727, 25380, 33445	54, 69, 130	—	—	—	—
[Cu(L)(NO_3_)_2_]	15432, 25575, 33670	55, 71, 135	—	—	—	—
[Cu(L)(CH_3_COO)_2_]	15290, 25380, 32570	53, 67, 130	—	—	—	—

**Table 4:** EPR spectral data of the Cu(II) complexes.

Complexes	${\text{g}}_{∥}$	g_*⊥*_	${\text{g}}_{\text{iso}}$	G
[Cu(L)Cl_2_]	2.10	2.03	2.05	3.34
[Cu(L)(NO_3_)_2_]	2.25	2.14	2.17	1.79
[Cu(L)(CH_3_COO)_2_)]	2.23	2.12	2.16	1.92

**Table 5:** Antibacterial screening data of the ligand and its Ni(II) and Cu(II) complexes.

Compounds	Diameter of inhibition zone (mm) (conc. in *μ*gml^−1^)					
	*Bacillus macerans*			*Pseudomonas striata*		
	250	125	63.5	250	125	63.5
Ligand(C_16_H_16_N_6_S_2_)	16	11	—	10	—	—
[Ni(L)Cl_2_]	22	16	—	20	14	8
[Ni(L)(NO_3_)_2_]	25	19	10	16	12	7
[Ni(L)(CH_3_COO)_2_]	18	10	—	15	9	—
[Cu(L)Cl_2_]	32	25	11	16	8	—
[Cu(L)(NO_3_)_2_]	28	16	10	18	12	9
[Cu(L)(CH_3_COO)_2_]	28	19	12	15	8	—
Streptomycin(standard)	35	26	14	28	20	12

**Table 6:** Antifungal screening data of the ligand and its Ni(II) and Cu(II) complexes.

Compounds	Fungal inhibition (%) (conc. in*μ*gml^−1^)								
	*Rhizoctonia batatiola*			*Alternaria alternata*			*Fusarium odum*		
	250	125	63.5	250	125	63.5	250	125	63.5
Ligand(C_16_H_16_N_6_S_2_)	50.2	29.3	11.2	56.2	30.2	11.2	48.2	22.0	—
[Ni(L)Cl_2_]	58.0	40.3	14.0	61.2	36.1	15.0	49.2	28.0	—
[Ni(L)(NO_3_)_2_]	52.2	32.1	12.2	57.0	34.2	12.0	51.0	23.4	11.2
[Ni(L)(CH_3_COO)_2_]	61.0	35.0	17.3	63.2	45.2	18.4	54.3	28.0	12.3
[Cu(L)Cl_2_]	76.3	48.0	35.0	79.0	48.0	22.0	65.0	32.0	14.0
[Cu(L)(NO_3_)_2_]	67.0	49.2	26.0	64.2	38.0	18.0	62.0	34.2	16.2
[Cu(L)(CH_3_COO)_2_]	70.1	45.3	28.0	59.3	33.0	12.0	62.2	30.0	12.2
Chlorothalonil (standard)	90.0	76.6	49.0	98.0	80.0	46.0	89.0	74.0	42.2
